# Crucial Role of TRPC1 and TRPC4 in Cystitis-Induced Neuronal Sprouting and Bladder Overactivity

**DOI:** 10.1371/journal.pone.0069550

**Published:** 2013-07-29

**Authors:** Mathieu Boudes, Pieter Uvin, Silvia Pinto, Marc Freichel, Lutz Birnbaumer, Thomas Voets, Dirk De Ridder, Rudi Vennekens

**Affiliations:** 1 Laboratory of Experimental Urology, Department of Development and Regeneration, KU Leuven, Leuven, Belgium; 2 Laboratory of Ion Channel Research, Department of Cellular and Molecular Medicine, KU Leuven, Leuven, Belgium; 3 TRP Research Platform Leuven, Leuven, Belgium; 4 Experimentelle und Klinische Pharmakologie und Toxikologie, Universität des Saarlandes, Homburg, Germany; 5 Pharmakologisches Institut, Universität Heidelberg, Heidelberg, Germany; 6 Laboratory of Neurobiology Signal Transduction, National Institute on Environmental Health Sciences, National Institutes of Health, Research Triangle Park, North Carolina, United States of America; University of Houston, United States of America

## Abstract

**Purpose:**

During cystitis, increased innervation of the bladder by sensory nerves may contribute to bladder overactivity and pain. The mechanisms whereby cystitis leads to hyperinnervation of the bladder are, however, poorly understood. Since TRP channels have been implicated in the guidance of growth cones and survival of neurons, we investigated their involvement in the increases in bladder innervation and bladder activity in rodent models of cystitis.

**Materials and Methods:**

To induce bladder hyperactivity, we chronically injected cyclophosphamide in rats and mice. All experiments were performed a week later. We used quantitative transcriptional analysis and immunohistochemistry to determine TRP channel expression on retrolabelled bladder sensory neurons. To assess bladder function and referred hyperalgesia, urodynamic analysis, detrusor strip contractility and Von Frey filament experiments were done in wild type and knock-out mice.

**Results:**

Repeated cyclophosphamide injections induce a specific increase in the expression of TRPC1 and TRPC4 in bladder-innervating sensory neurons and the sprouting of sensory fibers in the bladder mucosa. Interestingly, cyclophosphamide-treated Trpc1/c4^−/−^ mice no longer exhibited increased bladder innervations, and, concomitantly, the development of bladder overactivity was diminished in these mice. We did not observe a difference neither in bladder contraction features of double knock-out animals nor in cyclophosphamide-induced referred pain behavior.

**Conclusions:**

Collectively, our data suggest that TRPC1 and TRPC4 are involved in the sprouting of sensory neurons following bladder cystitis, which leads to overactive bladder disease.

## Introduction

The bladder-innervating afferent fibres (A∂ and C) are activated during bladder filling and transmit information to the brain about the degree of bladder distension and the amount of urine stored in the bladder [1], [2].

Overactive bladder (OAB) syndrome is the common cause of urinary incontinence, the involuntary leakage of urine. This distressing condition has a major impact on quality of life and on healthcare resources [3]–[6]. The mechanisms underlying OAB are not fully understood. OAB is a clinical diagnosis featured by objective detrusor (bladder muscle) overactivity, which can occur in spinal cord injury patients or in patients with bladder inflammation (cystitis). Both in patients and in animal models cystitis a deep remodeling of bladder-innervating neurons has been observed, including proteome modification [7], [8], sensitization of ion channels and increased neurotrophic factors release [9]. Moreover, bladder inflammation induces hyperexcitability of DRG accompanied with an increased somata size and enhances afferent innervations [10]–[13]. This hyperinnervation suggests the occurrence of sprouting of peripheral nerves during chronic cystitis, which may in turn lead to increased signaling from the bladder to the spinal cord, and therefore play a key role in the urgency symptoms of cystitis [13].

Transient receptor potentials (TRP) channels exhibit sensitivity to a large variety of sensory stimuli, and are therefore generally considered as cellular sensors. TRP channels are classified in six subfamilies: TRPV (vanilloid), TRPC (canonical), TRPA (ankyrin), TRPM (melastanin), TRPML (mucolipin) and TRPP (polycystin). Bladder-innervating sensory neurons are known to express TRPA1 [14], TRPM8 [15] and TRPV1 [16], but to date the expression or function of TRPCs in bladder-innervating neurons has not been documented. TRPC channels are most often described as Ca^2+^-permeable non-selective cation channels activated downstream of receptor stimulation [17]. Adult sensory neurons are known to express five members of the family (TRPC3> TRPC6> TRPC1> TRPC4> TRPC5) [18]. TRPC channels may be particularly interesting in the framework of hyperinnervation of the bladder, as several TRPCs have been implicated in neuronal cell growth, remodeling, axon guidance and growth cone signaling [19]. For example, TRPC4 and TRPC5 modulated axon growth in DRG post injury and in hippocampus, respectively [20], [21]. Moreover, TRPCs mediate Ca^2+^ influx and subsequent growth cone turning response to netrin-1 and BDNF [22], [23].

Here, we show increased expression of TRPC1 and TRPC4 in bladder-innervating sensory neurons, and provide evidence for their involvement in afferent sprouting and concomitant bladder overactivity in the chronic cyclophosphamide (CYP) model of cystitis.

## Materials and Methods

### Animals

Experiments were conducted on 8–12 weeks female Wistar rats and on 8–10 weeks old Trpc1^−/−^, Trpc4^−/−^ and Trpc1/Trpc4^−/−^ mice [24], [25]. Animals were housed in an animal house with a 12-hour light-dark cycle and allowed water and standard food *ad libitum*.

### Ethics Statement

All experiments approved by the KU Leuven Ethical Committee Laboratory Animals (ECD). Approval was granted under project number P089/2011. The authors’ laboratory has the Belgian Governmental license for small animal experiments (LA1210551).

### Cystitis Induction

Cyclophosphomide (CYP)-induced cystitis was provoked by four i.p injections every second day for seven days (40 mg/kg; chronic)[26]–[28]. Controls animals were injected with four saline injections. Bladder inflammation was confirmed both morphologically, using hematoxylin and eosin staining, and functionally, using cystometry, as previously described [28]. We did not observe different inflammation state in the bladder of the different mice strains (Figure S1).

### Retrograde Labeling of Bladder Neurons

Rats were anesthetized with 3% inhaled isoflurane, the bladder was exposed and 5 µl of 1,1′-dioctadecyl-3,3,3′3′-tetraethylindocarbocyanine perchlorate [DiIC_18(3)_] (Invitrogen; 0.2 mg/ml in DMSO) was injected into five sites within the bladder wall and around the trigone using a 10 µl syringe (SGE). The syringe was kept in place for 30 seconds to avoid leakage, and saline solution was used to rinse the operation field between each injections. The wound was sutured and postoperative analgesia was provided after surgery (30 µg/kg i.p of Temgesic; Schering-Plough Animal Health). DRG were sampled 10 days after dye injection.

### Quantitative Retro-transcription PCR

Total RNA was extracted from lumbar L6 and sacral S1 DRG of control and cyclophosphomide injected adult rats using the RNeasy Mini Kit (Qiagen) and subsequently served for cDNA synthesis using Superscript III (Invitrogen). Quadriplicates of four independent experiments were analyzed by quantitative real-time polymerase chain reaction (qPCR) in the 7500 Real Time PCR system (Applied Biosystems) using specific TaqMan gene expression assays for all TRP channels (Applied Biosystems). The two reference genes were polymerase (RNA) II polypeptide J and DEAD box polypeptide 48 [29].

### Immunohistochemistry

#### DRG staining

Frozen transverse sections (14 µm thick) were prepared from adult lumbar L6 and sacral S1 DRG fixed with 4% paraformaldehyde in PBS and cryopreserved in 25% sucrose in PBS, before embedding in O.C.T. compound (Sakura Finetek, Zoeterwoude, The Netherlands). The sections were incubated with 10% goat serum in PBT 0.1% for 1h at room temperature. Slides were then incubated 2h at room temperature with the TRPC1 (Alomone Lab) [30], TRPC4 (Chemicon) [31], or GAP-43 (Millipore) primary antibodies (1∶500 in PBT 0.3% with 1% goat serum). After washing the primary antibody with PBT 0.1%, sections were incubated for 1 h at room temperature with anti-rabbit secondary antibody (1∶500 in PBT 0.1%). The slides were then washed in PBS before mounting with Fluorsave medium. As control, we tested both TRPC1 and TRPC4 antibodies by using Trpc1/c4^−/−^ DRG. The TRPC1 antibody specifically stained the WT DRG, whereas some nonspecific staining was observed using TRPC4 antibody (Fig. S2), but unlikely to explain the pattern expression observed in control and CYP_c_ conditions.

Images were collected using a LSM510 confocal microscope (Zeiss). Images were acquired using Axio Vision (Zeiss) and analyzed with Image J.

### Mucosa Whole Mount Staining

Bladders were harvested, cut opened, stretched out and fixed for 4h at 4°C with formamide. The mucosa was gently harvested from the body of the bladder with forceps (excluding muscle fibers from the preparation), washed in PBS and incubated sequentially for 2 h with PBT0.3% +20% serum, overnight at 4°C with primary antibodies (PGP9.5 (Millipore - 1∶500), Tuj1 (Millipore - 1∶1000), VAChT (Santa Cruz –1∶500) and CGRP (Sigma - 1∶500)) in PBT0.3% +5% serum and for 2 h at room temperature with secondary antibodies at 1∶500. The mucosa was then washed in PBS before mounting with Fluorsave medium. This protocol has been modified from [32]. Images were collected using an Apotome microscope (Zeiss) and acquired using Axio Vision (Zeiss).

### Neurite Quantification

Fibres identified visually as longer than 10 µm with a thickness between 1 and 3 µm, positive to PGP9.5 were considered as neurites. The neuronal nature of the fibres was confirmed by co-staining with PGP9.5 and Tuj1 in rat mucosa whole mount. We analyzed the images with ImageJ software and we quantified neurites on 8–10 images per bladder from 3–4 animals per condition. Results were expressed as neurite segments/µm^2^ (number of individual neurite per surface unit), the neurite length and the neurite density (neurite length per surface unit).

### Cystometry

A full description of the cystometry assay can be found in Uvin et al [33]. Briefly, a PE-100 50 polyethylene catheter (Becton Dickinson) with a cuff was inserted in the bladder dome via an abdominal midline incision, and fixed using a 6/0 monofilament polypropylene purse-string suture (Ethicon). The catheter was flushed with saline to exclude leakage and then tunneled subcutaneously to the neck. Finally the abdominal wall and skin were closed. The cystometry was done in restrained and conscious animals. The bladder catheter was connected to a pressure transducer (Becton Dickinson) and an infusion pump (Harvard Apparatus) via a T tube. The pressure transducer output was amplified and recorded using a Windaq data acquisition system (Dataq Instruments). Bladders were filled at constant rate (100 µl/min in rats and 20 µl/min in mice) and after a 30 min equilibration period, intravesical pressure was recorded for 30 min. Individual urine voids were collected with a balance.

#### Muscle strips contractility

Animals were sacrificed; bladders were excised and placed in Krebs’ solution (in mM) (NaCl 118, KCl 4.6, CaCl_2_ 2.5, MgSO_4_ 1.2, KH_2_PO_4_ 1.2, NaHCO_3_ 25, glucose 11). The muscle strip was weighed, placed in an organ bath and attached to a steel hook at one end and to a fixed force transducer at the other end (Harvard Apparatus, Holliston, MA) to enable isometric measurements. Krebs’ solution was constantly gassed with a mixture of O_2_ (95%) and CO_2_ (5%) and the temperature was maintained between 35 and 37°C with a pH of 7.40. Contractility was measured as tension changes, sampled using homemade software that was driving two of the four A/D channels of a Dalanco D310 DSP board (Dalanco Spry, Rochester, NY) and connected to the transducers. During the accommodation period (30 minutes) we aimed to create a similar pre-tension on all bladder strips, obtained by an initial preload of 1 g, and subsequent readjustments were made every 10 min until this preload remained constant. Each experiment consisted of 5 min of rest, followed by adding carbachol (10 nM) directly to the bathing solution or replacement of the bathing solution with high KCl solution (122 mM).

#### Mechanical sensitivity testing

The mechanical sensitivity was assessed with Von Frey filaments. Following a habituation period of 2 h, mice were tested in individual cage with stainless steel wire grid floor. Stimulation was confined to the lower abdominal area in the general vicinity of the bladder, and care was taken to stimulate different areas within this region to avoid desensitization. Two types of behavior were considered as positive responses to filament stimulation: sharp retraction of the abdomen and immediate licking or scratching of the stimulation area. Each filament was applied for 1–2 sec with an interstimulus interval of 5 sec. We used the “up and down” method [34] to determine the 50% threshold (T_50_). According to [35] the calculation of the most reliable threshold requires six successive tries with different filaments. The formula T_50_ =  Xf+kd (Xf, the last filament used; k, the Dixon’s table coefficient and d the average of the filament interval) was applied to determine T_50_.

### Statistical Analysis

All values are reported as mean ± SEM. Comparisons of the means observed in different groups were performed using a Student’s *t* test or ANOVA, as appropriate (Graphpad Prism 5). *p<0.05; **p<0.01 and *** p<0.001.

## Results

### Afferent Nerve Network is Denser in Chronic CYP-treated Rats

**Figure 1:**
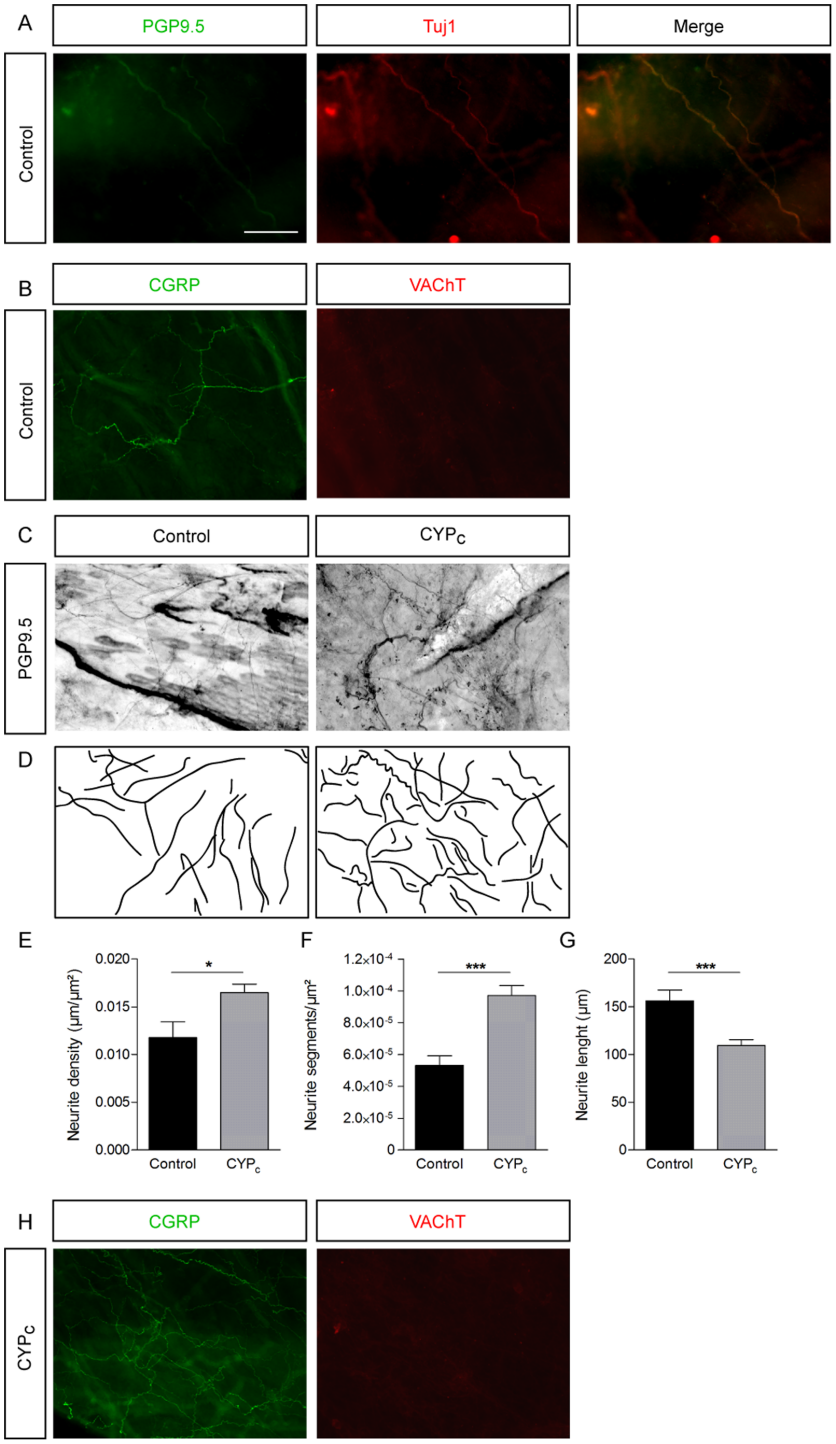
Sensory neurites sprouting in bladder wall in CYPc rats. *****A*,**** Nerve fibers stained for PGP9.5 and Tuj1 in whole mount urothelium - scale bar : 50 µm. **B,** Fibers are stained by CGRP antibody but not by VAChT antibody in control condition. ***C,*** PGP9.5 staining of whole mount bladder mucosa in control and CYP-treated rats. **D,** Line representation showing neurites based on the images in panel **C**, illustrating increased outgrowth in CYP_c_. **E-G,** Statistical comparison of neurite segments (**E**), neurite length (**F**) and neurite density (**G**) between control and CYP_c_ rats. **H**, Neurite fibers observed in CYP_c_ are stained by CGRP but by VAChT antibody.

In whole mount stained mucosas, we identified nerve fibers by the colocalization of PGP9.5 and Tuj1 (Fig. 1A). These fibers were positive for CGRP and negative for the vesicular acetylcholine transporter (VAChT), indicating that they represent sensory nerves rather than motor nerves [36](Fig. 1B). Staining of peripheral neurites with PGP9.5 antibody in whole mount mucosas revealed an increase in bladder innervation in CYP-treated rats (CYP_C_) (Fig. 1C, D). Analysis of the nerve distribution pattern revealed an increased branching (9.71.10^−5^±0.06.10^−5^ segment/µm^2^ in CYP_C_ compared to 5.35.10^−5^±0.63.10^−5^ segment/µm^2^ in control conditions; p<0.001; n  = 9 and 8 in control and CYP_c_ conditions from 3 bladders)(Fig. 1F) and a decrease of the length of individual neurite segments (from 156.3±11.3 µm to 109.6±5.6 µm in CYP_c_; p<0.001) (Fig. 1G). The total density of neurites increased by 33% in CYP_c_ mucosas compared to controls (respectively, 0.016±0.001 µm/µm^2^ and 0.012±0.002 µm/µm^2^; p<0.05)(Fig. 1H). We also observed the sprouting of CGRP positive fibers but no appearance of VAChT antibody staining (Fig. 1H). Taken together, these results demonstrate an increased afferent innervation in mucosas of CYP_c_ rats.

### The Growth Associated Protein 43 is Overexpressed in DRG of CYP_c_ Rats

**Figure 2:**
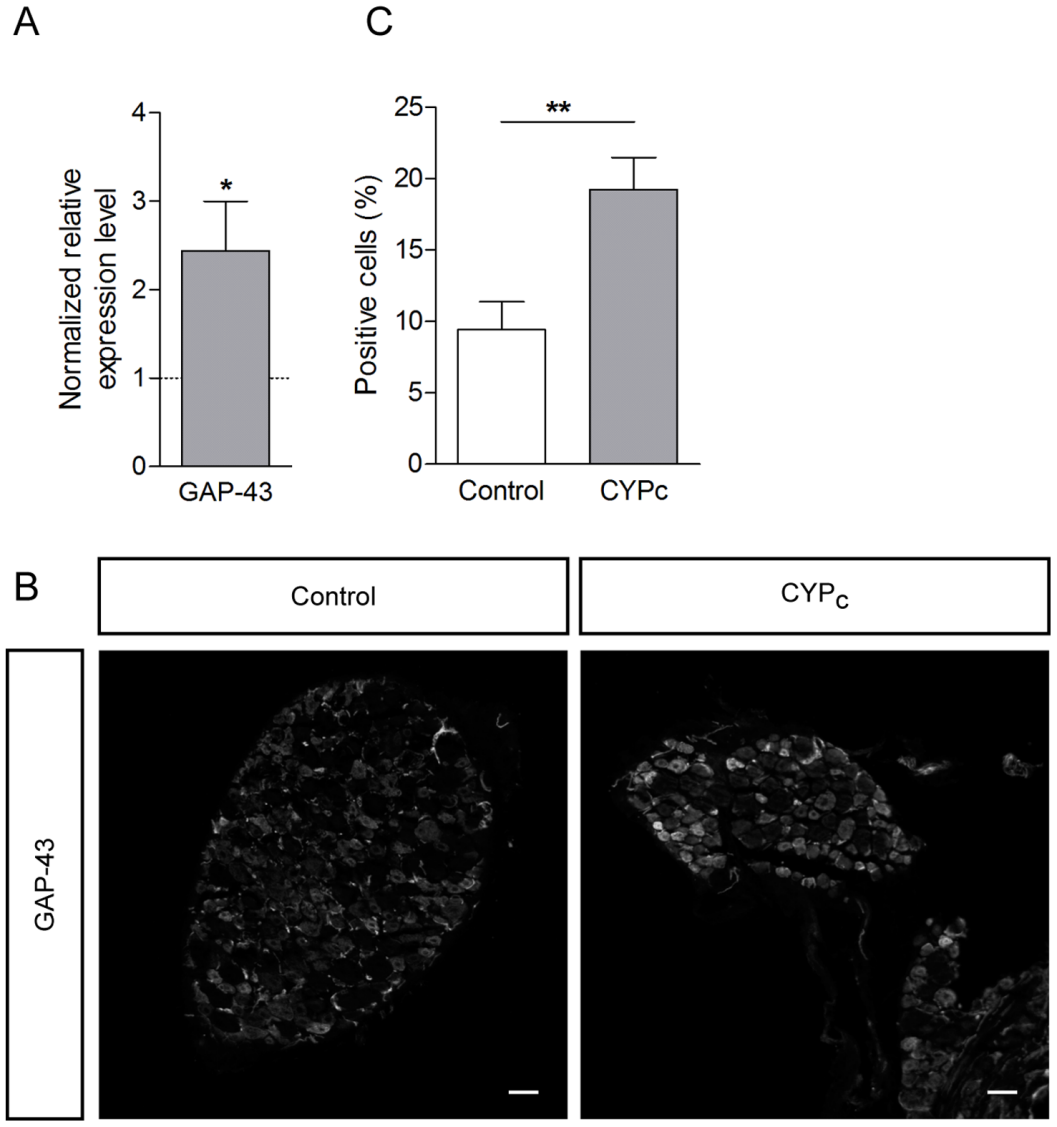
Cyclophosphamide treatment induces overexpression of GAP-43 in DRG. **A,** qRT-PCR analysis of GAP-43 transcripts in L6-S1 DRG shows a significant increase of GAP-43 expression after CYP treatment. **B,** Neurite outgrowth marker GAP-43 staining in DRG of control and CYP_c_ rats. **C,** Quantification of number of GAP-43 positive cells in bladder-innervating DRG - scale bar: 50 µm.

We confirmed the outgrowth of peripheral neurites in the mucosa by the analysis of the expression of GAP-43, a growth cone protein related to neurite outgrowth [37]. We found that GAP-43 transcripts were 2.4-fold more abundant in total DRG in CYP_c_ compared to controls (p<0.05)(Fig. 2A). Moreover, the number of GAP-43-positive neurons was 9.4±1.9% in control and increased to 19.2±2.2% in CYP_c_ (p<0.01)(Fig. 2B and 2C). The data confirmed that the hyperinnervation is associated with nerve growth in chronically treated rats.

### CYP_c_ Rat Bladder is not Innervated by More Bladder Afferent Neurons

**Figure 3:**
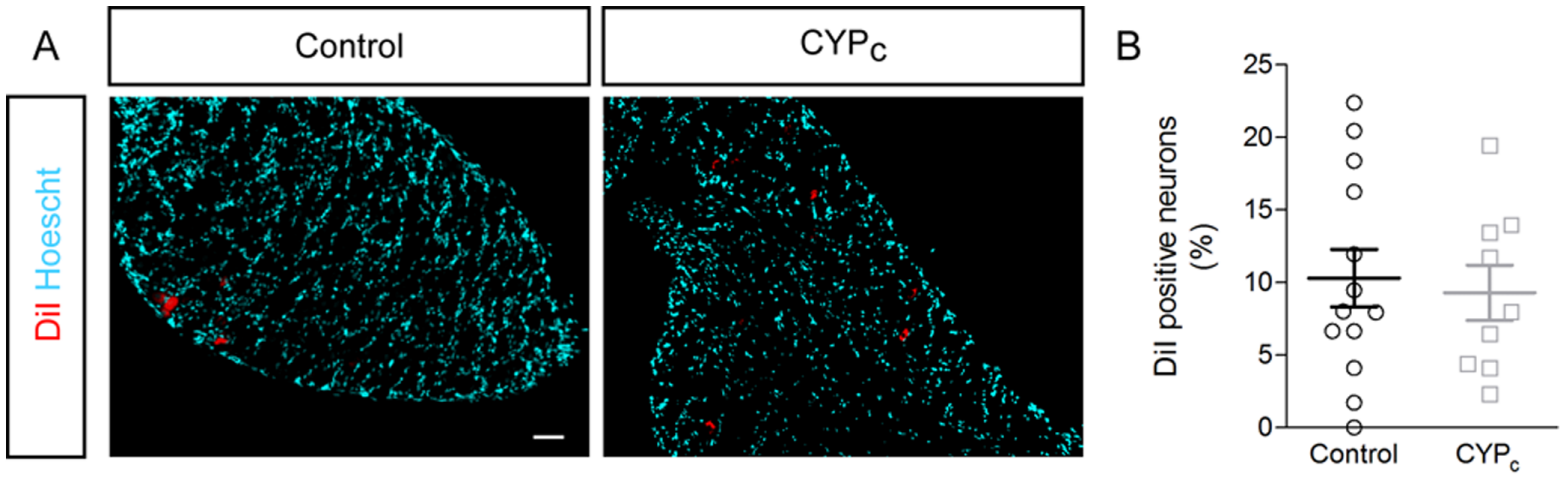
CYP_c_ bladders are not innervated by more sensory neurons. **A,** Imaging of retrolabelled bladder neurons with DiI dye - scale bar: 50 µm. **B,** The quantitative analysis shows that bladder is not innervated by more afferent neurons.

We retrolabelled bladder sensory neurons by the injection of DiI into the bladder wall and ten days later, DRG were fixed and sliced to observe fluorescence, as described previously [14](Fig. 3A). We did not observe any change in the number of retrogradely labeled neurons in CYP_c_ (9.3±1.9% neurons/slice, n  = 9 in CYP_c_ DRG; compared to 10.3±1.9% neurons/slice, n  = 13 in control DRG; p>0.05) (Fig. 3B), indicating that hyperinnervation of CYP_c_ mucosas is not due to an increased recruitment of neurons from L6 and S1 DRG.

### TRPC1 and TRPC4 are Overexpressed in Bladder-innervating DRG Neurons in CYP-Treated Rats

**Figure 4:**
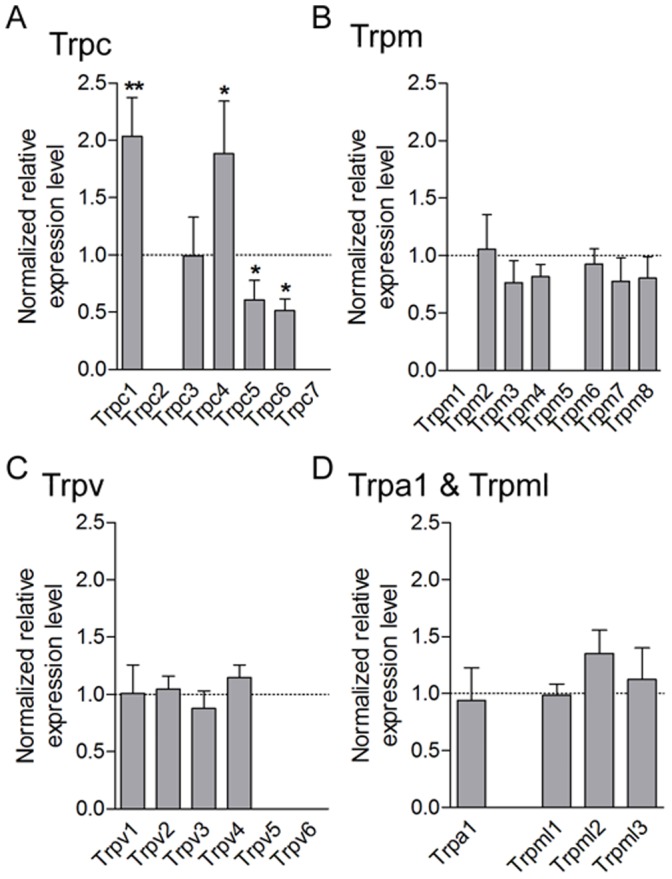
TRP expression screening in DRG of CYPc rats. **A–D,** qRT-PCR analysis of Trpc (**A**), Trpm (**B**), Trpv (**C**), and Trpa1 and Trpml (**D**) mRNA shows an up-regulation of Trpc1 and Trpc4 transcripts and a decreased expression of Trpc5 and Trpc6 transcripts in L6-S1 DRG of CYP-treated rats.

To examine whether the induction of chronic cystitis was accompanied by specific changes in TRP channel transcript levels, we compared expression of TRPA, TRPCs, TRPMs, TRPMLs and TRPVs members in L6-S1 DRG from control and CYP_c_ animals using quantitative RT-PCR. Among all TRP channels expressed in DRG, only TRPC1 and TRPC4 mRNA was increased in CYP_c_ rats compared to control rats, respectively (2.36±0.34, p<0.01, n  = 8 and 1.89±0.46, p<0.05, n  = 8) (Fig. 4A). We also noticed a significant decrease in the expression of TRPC5 and TRPC6 (p<0.05) (Fig. 4A). The relative expression levels of TRPM, TRPV, TRPA and TRPML channels were not significantly modified in CYP_c_ rats (Fig. 4B, 4C, 4D)(p>0.05).

**Figure 5:**
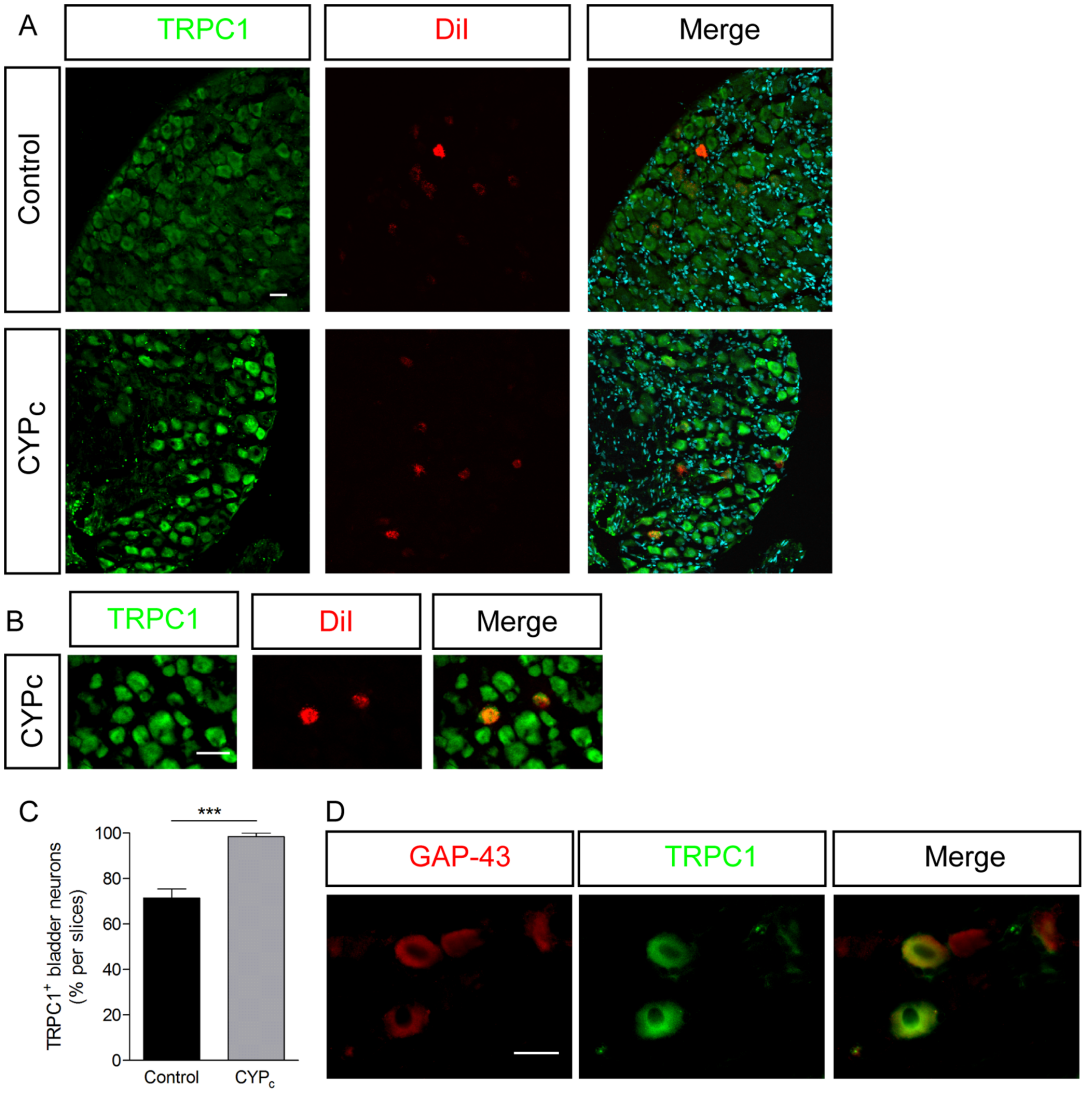
TRPC1 is upregulated in bladder-innervating neurons in CYP_c_ rats. **A, B** Immunocytochemistry of TRPC1 in DRG of control and CYP_c_ rats, retrolabelled with DiI - scales bar: 50 µm. **C,** Quantitative analysis of colocalization of DiI and TRPC1. **D,** Immunohistochemistry showing that a majority of GAP-43 positive neurons are TRPC1 positive in CYP_c_ rats – scale bar: 20 µm.

**Figure 6:**
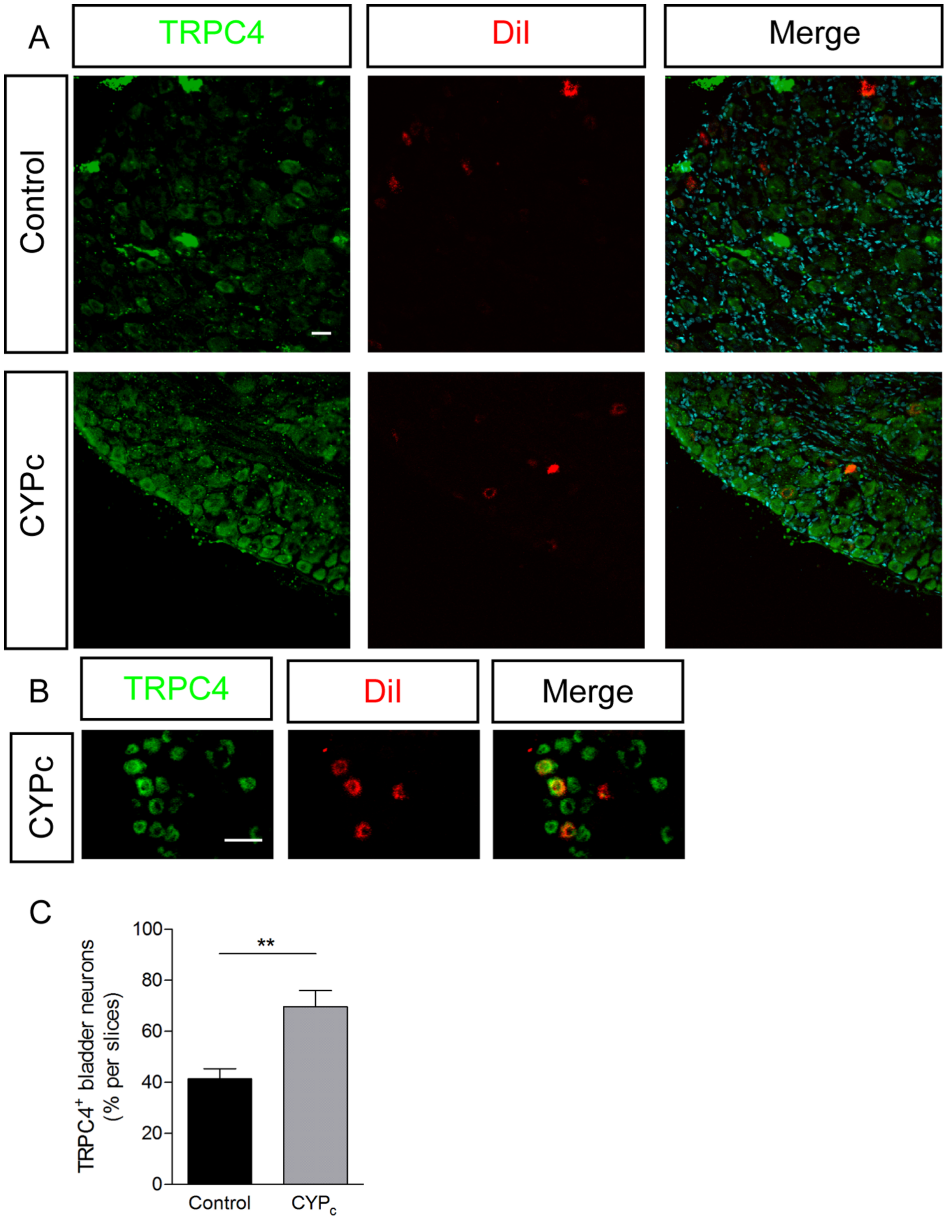
TRPC4 is upregulated in bladder-innervating neurons in CYP_c_ rats. **A, B** Immunocytochemistry of TRPC4 in DRG of control and CYP_c_ rats, retrolabelled with DiI - scales bar: 50 µm. **C,** Quantitative analysis of colocalization of DiI and TRPC4.

In line with these PCR data, we found that the number of bladder-neurons that reacted with antibodies raised against TRPC1 (Fig. 5) and TRPC4 (Fig. 6) was significantly increased in DRG from CYP_c_ rats. The vast majority of the growing, GAP-43 positive neurons was also positive to TRPC1 (Fig. 5D)(84/103; 81.5%). Taken together, these results indicate that the CYP-induced cystitis in rats induces the *de novo* expression of TRPC1 and TRPC4 in bladder sensory neurons.

### TRPC1 and TRPC4 Inhibition Decreases Sprouting Induced by Chronic Administration of CYP

To assess a possible involvement of TRPC1 and/or TRPC4 in the process of neurite sprouting in cystitis, we compared changes in CYP-treated wild type mice and mice deficient in TRPC1, TRPC4 and TRPC1/C4 (Trpc1^−/−^, Trpc4^−/−^ and Trpc1/c4^−/−^ mice).

**Figure 7:**
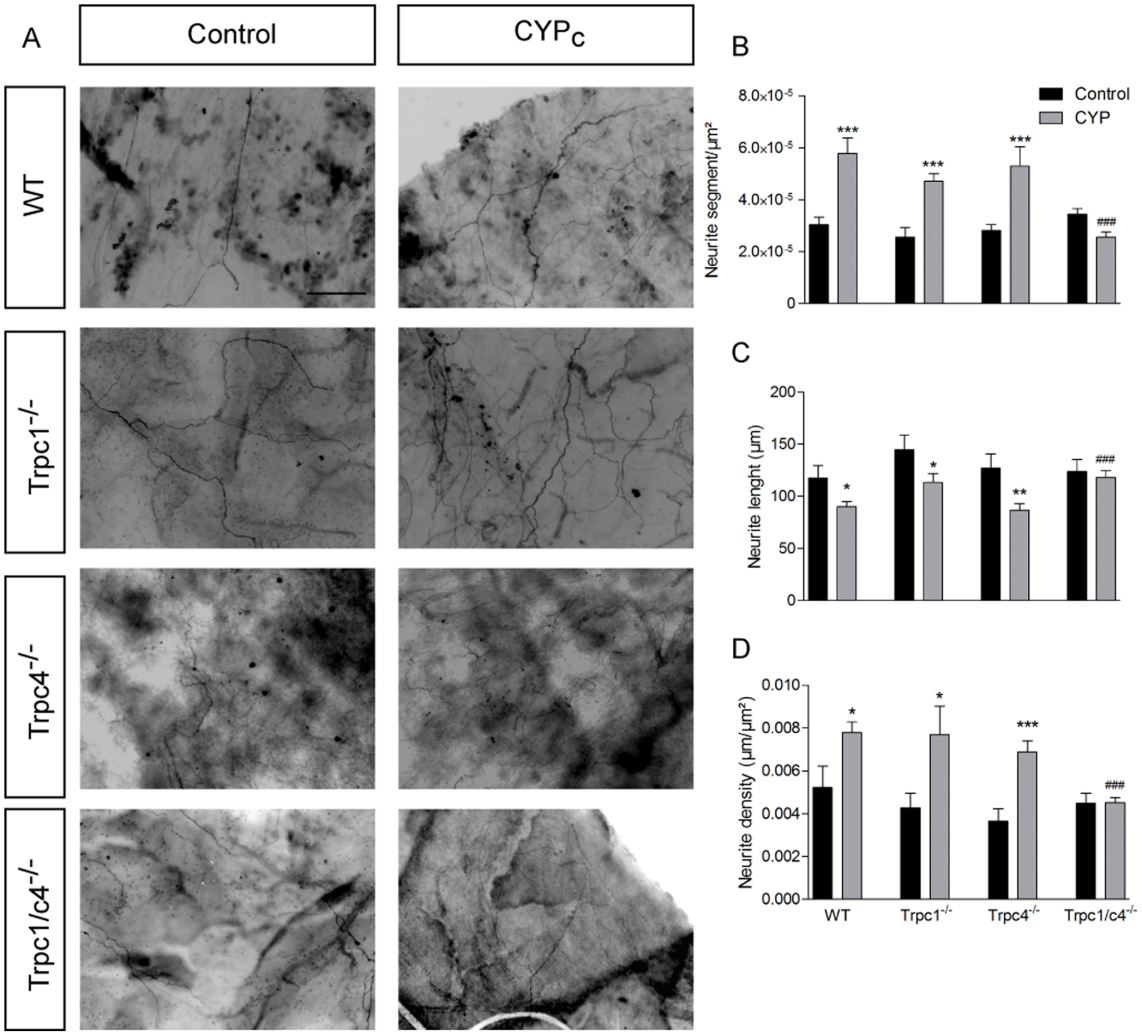
CYP-induced peripheral sprouting is abolished in Trpc1/c4^−/−^ mice. **A,** PGP9.5 staining in whole mount bladder mucosa in control and CYP-treated wild type, TRPC1, TRPC4 and TRPC1/C4 deficient mice - scale bar : 50 µm. **B–D,** Quantitative analysis of neurite segments (**B**), neurite length (**C**) and neurite density (**D**) shows a normal innervation in treated Trpc1/c4^−/−^ mice.

In vehicle-treated animals, we did not observe any significant difference in the innervation pattern of the bladder between wild type, Trpc1^−/−^, Trpc4^−/−^ and Trpc1/c4^−/−^ mice. Like in rats, we found that CYP treatment in mice caused a striking increase in the number of neurite segments and density of neurites and a decrease of the average neurite length. Similar CYP-induced changes in innervation were observed in Trpc1^−/−^ and Trpc4^−/−^ mice. In contrast, CYP treatment did not increase bladder innervations in Trpc1/c4^−/−^ mice (Fig. 7). These results support the hypothesis that TRPC1 and TRPC4 are responsible for the neuronal sprouting in the inflamed bladder.

### Inhibition of CYP-induced Sprouting Ameliorated Overactive Bladder Phenotype

**Figure 8:**
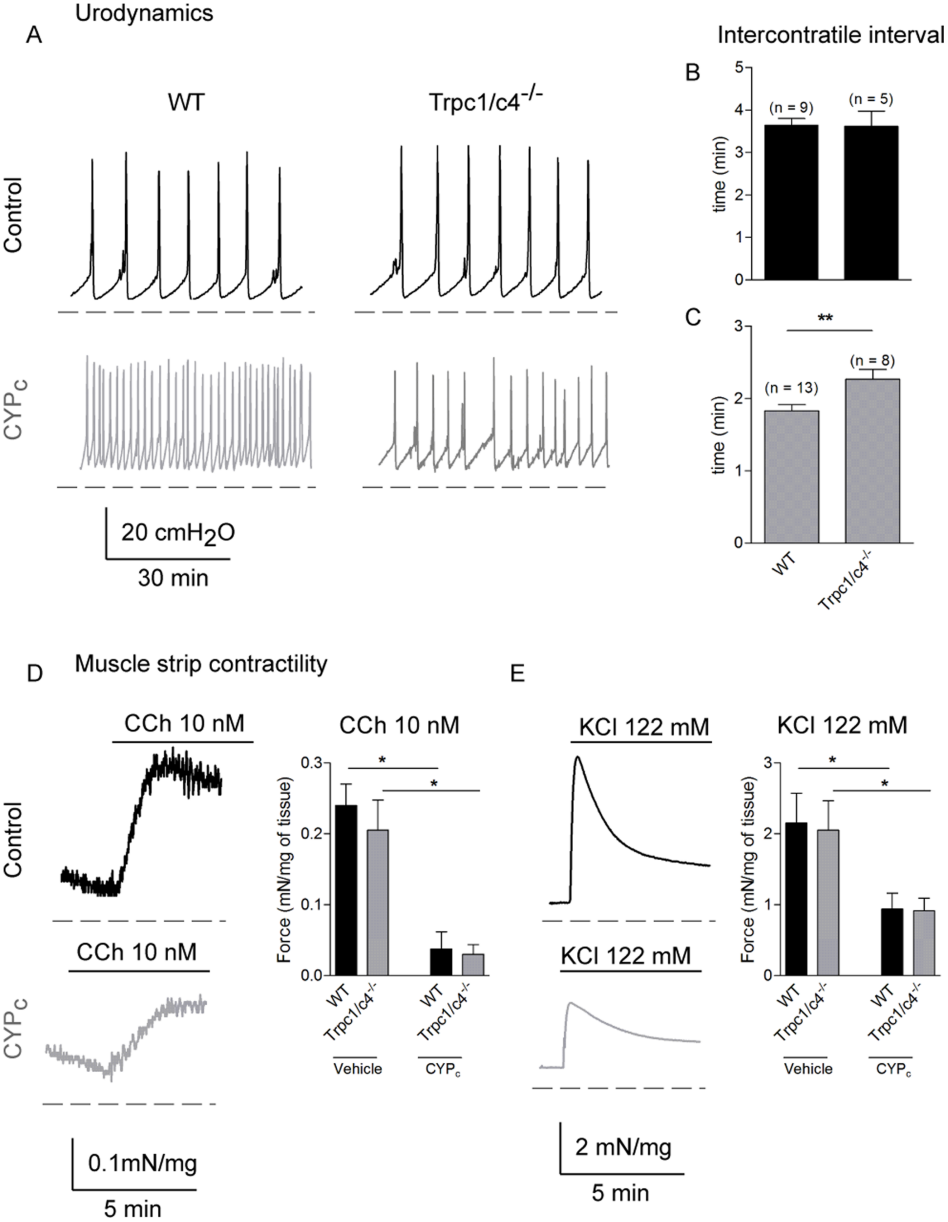
CYP-induced bladder overactivity is decreased in Trpc1/c4^−/−^ mice. **A,** Typical urodynamic traces of WT and Trpc1/c4^−/−^ mice in vehicle or following CYP treatment. **B,C,** Analysis of intercontractile interval in vehicle (**B**) and CYP-treatment mice (**C**) in WT and Trpc1/c4^−/−^ mice shows that double knock-out mice are functionally less affected by CYP treatment. **D,** Typical muscle strip contractility traces of WT mice in control and in CYP_c_ rats activated by 10 nM of carbachol. The statistical analysis of carbachol-induced contraction of muscle strips did not show difference between the two genotypes in CYP_c_ mice. **E,** Typical traces of muscle strip contractility of WT mice stimulated with high KCl concentration (122 mM). There is no difference between WT and Trpc1/c4^−/−^ in control and CYP_c_ conditions.

To test whether nerve sprouting played a role in the CYP-induced bladder overactivity, we performed cystometry in vehicle- and CYP-treated wild type and Trpc1/c4^−/−^ mice. The urodynamic hallmark of bladder overactivity is the increase in void frequency when saline solution is infused at a constant rate into the bladder. The cystometry measures the intravesical pressure and each peak indicates bladder contraction and leads to voiding of urine. The most relevant parameter to assess bladder overactivity is the intercontractile interval. Following vehicle treatment, no significant differences were found between genotypes in urodynamic pattern (respectively in WT and Trpc1/c4^−/−^; intercontractile interval: 3.6±0.2 min and 3.6±0.3 min; p>0.05; baseline: 4.2±0.3 cmH_2_0 and 5.0±0.3 cmH_2_0; p>0.05; voiding volume: 72.8±7.2 µl/void and 72.2±9.1 µl/void; p>0.05; n  = 9 WT and 5 Trpc1/c4^−/−^ mice)(Fig. 8A and 8B). In line with previous work, chronically injected CYP induced bladder overactivity in WT (Fig. 8A). However, following treatment bladder overactivity was less pronounced in Trpc1/c4^−/−^ mice with a longer intercontractile interval (Fig. 8A, 8C) whereas the baseline pressure (8.4±0.4 cmH_2_0 and 7.7±0.2 cmH_2_0, p>0.05) and the amplitude (37.6±1.3 cmH_2_0 and 35.2±1.6 cmH_2_0) were not modified.

Although expression of TRPC1 and TRPC4 has never been reported in the detrusor, TRPC1 and TRPC4 were detected with immunohistochemistry respectively in the urothelium and suburothelium [31]. To investigate a possible contribution of TRPC1 and TRPC4 to detrusor physiology, we performed isolated muscle strip contractility measurements. We did not observe any difference between the WT and the Trpc1/c4^−/−^ strips force in controls and CYP_c_ conditions when stimulated with carbachol (10 nM) or a depolarizating KCl (122 mM) solution (Fig. 8D, 8E).

CYPc mice exhibited an increased referred hyperalgesia [28]. We tested whether a reduced sprouting induced a decreased pain behavior with Von Frey filament. The pain threshold was not different between WT and Trpc1/c4^−/−^ mice in control conditions (respectively 0.09±0.04 g; n  = 6 and 0.10±0.05 g; n  = 6; p>0.05). The threshold-decreased amplitude was not statistically modified in CYP_c_ WT and Trpc1/c4^−/−^ (−73.5±9.3% and −79.9±5.4%; p>0.05).

We can conclude that bladder function of Trpc1/c4^−/−^ mice is less altered following chronic CYP treatment.

## Discussion

Here we have presented several lines of evidence supporting a previously unrecognized role for TRPC1 and TRPC4 in the development of bladder overactivity, as crucial mediators of sprouting and nerve outgrowth of sensory neurons innervating the bladder wall. We show (1) that sprouting of afferent neurons is a hallmark of CYP-induced cystitis in rats and mice, without recruitment of new fibres, (2) that the expression of TRPC1 and TRPC4 is increased in bladder-innervating sensory neurons of CYP-treated animals, (3) that double knock-out mice (Trpc1/c4^−/−^) fail to develop increased innervation of the bladder wall following CYP-treatment, and (4) finally that CYP-induced bladder overactivity is less pronounced in these Trpc1/c4^−/−^ mice.

A sensory signal originating from the bladder is processed by the central nervous system and in a socially convenient time and space, the detrusor contracts and the sphincter relaxes to expel urine. Afferent activity is mainly triggered by A∂ and C-fibres. They express a wide variety of ion channels among them TRP channels [38], which are potential novel pharmacological targets to treat overactive bladder [39]. Many groups reported morphological changes in neuron size, excitability and hyperinnervation in overactive bladder patients [10]–[13]. Moreover, in an acute CYP-induced overactive bladder, Dikson and colleagues described a peptidergic and parasympathetic fibres sprouting in the mucosa [40]. We recently standardized and characterized a chronic CYP-induced overactive bladder model in mice that is closely related to human pathology [28]. We firstly analyzed cystometry of treated rats with the same protocol and showed that mice and rats urodynamics features were similar. We previously showed that cultured urothelium cells released more NGF in CYP-treated conditions [28] and that might trigger the upregulation of TRPC1 and TRPC4 genes in DRG as neutrophins are known to regulate TRP gene expression in murine mucosa [41] and urothelium (Schnegelsberg et al., 2010).

Our study highlights a crucial role of both TRPC1 and TRPC4 in bladder-afferent nerve sprouting and in bladder dysfunction in a chemically induced overactive bladder model. Our qPCR data indicate increased expression of TRPC1 and TRPC4 mRNA in DRG neurons following CYPc treatment. Although some antibody aspecificity cannot be fully excluded, immunohistochemistry using previously described anti-TRPC1 and anti-TRPC4 antibodies [30], [31] confirmed the increased expression of TRPC1 and TRPC4 in DRG neurons, and more specifically in neurons innervating the bladder. TRPC1 and TRPC4 are able to form heterotetrameric channels with properties that differ from TRPC1 and TRPC4 homotetramers [42], at least in vitro. We found that Trpc1/c4^−/−^ mice showed no increase in the innervations density of the bladder wall following CYP-treatment, whereas the individual single knockout mouse strains (Trpc1^−/−^ and Trpc4^−/−^ mice) developed the same degree of hyperinnervation as the wild type mice. This speaks against a crucial role for TRPC1-TRPC4 heteromultimeric channels in bladder-innervating neurons, and rather suggests that TRPC1 and TRPC4 have overlapping functions and can compensate for each other’s loss in the single knockout mice. We cannot rule out compensatory mechanism to explain the lack of phenotype in Trpc1^−/−^ and Trpc4^−/−^ mice. However, earlier studies did not report compensation by other TRPs or ion channels in other systems [43], [44].

TRPC channels have been previously implicated in neurite growth and chemotrophic guidance of neuronal endings by regulating calcium concentration in the growth cone [21], [23]. Interestingly, in hippocampal neurons, overexpression of dominant negative TRPC5 leads to significantly longer neurite suggesting an inhibitory role for the channel [20]. In this regard, we noticed a decrease TRPC5 expression in bladder-afferent neurons following CYP-treatment in rat which might suggest a similar role in those neurons.

We found no evidence for a specific role of TRPC1 and TRPC4 in detrusor function in control and CYP_c_ condition, as muscle strips from Trpc1/c4^−/−^ behaved similarly upon carbachol and high K^+^ stimulation. This further supports the contention that the cystometric phenotype observed in double knock mice is related to altered sprouting of sensory fibers rather than to changes in bladder smooth muscle functioning. The afferent innervation in bladder mucosa also contributes to the mechano- and chemosensation of bladder. Increased innervation is therefore expected to affect bladder function by increased sensory detection capabilities. On the one hand, more neurites in bladder mucosa results in an increase in P2X3 density [13], which might lead to the afferent hyperexcitability [2] upon an enhanced ATP release in CYP-treated rodents [45].

As mentioned earlier, L6-S1 DRG innervate diverse organs (i.e. colon, skin [46], [47] and single bladder-innervating neurons may also have branches innervating the skin [46]. Moreover, cystitis induces cross-sensitization of DRG neurons [47]. In this respect, it was somewhat surprising that the Trpc1/c4^−/−^ mice developed the same degree of mechanical hyperalgesia of the lower abdomen following CYP treatment as wild type mice. It should be noted, however, that specific visceral pain is difficult to quantify and mainly assessed as a globally increased abdominal sensitivity. Therefore, we cannot fully exclude a contribution of TRPC1 and TRPC4 to CYP_c_-induced bladder pain.

In conclusion, our data indicated that TRPC1 and TRPC4 are important players in the development of bladder dysfunction in the CYP-induced overactive bladder, by promoting bladder-afferent sprouting in mucosa. Further studies will be necessary to understand the underlying mechanisms by which TRPC1 and TRPC4 promote *de novo* nerve growth in murine cystitis.

## Supporting Information

Figure S1 Histology of bladder from WT and Trpc1/c4^−/−^ mice in control and CYP_c_ conditions. (TIF)

Figure S2 TRPC1 and TRPC4 antibodies specificity test in DRG WT and Trpc1/c4^−/−^. (TIF)
