# Supplementary figures and images for: Crucial Role of TRPC1 and TRPC4 in Cystitis-Induced Neuronal Sprouting and Bladder Overactivity
Source: PLoS One. 2013 Jul 29;8(7):e69550. doi: 10.1371/journal.pone.0069550 (PMC3726658; doi:10.1371/journal.pone.0069550)

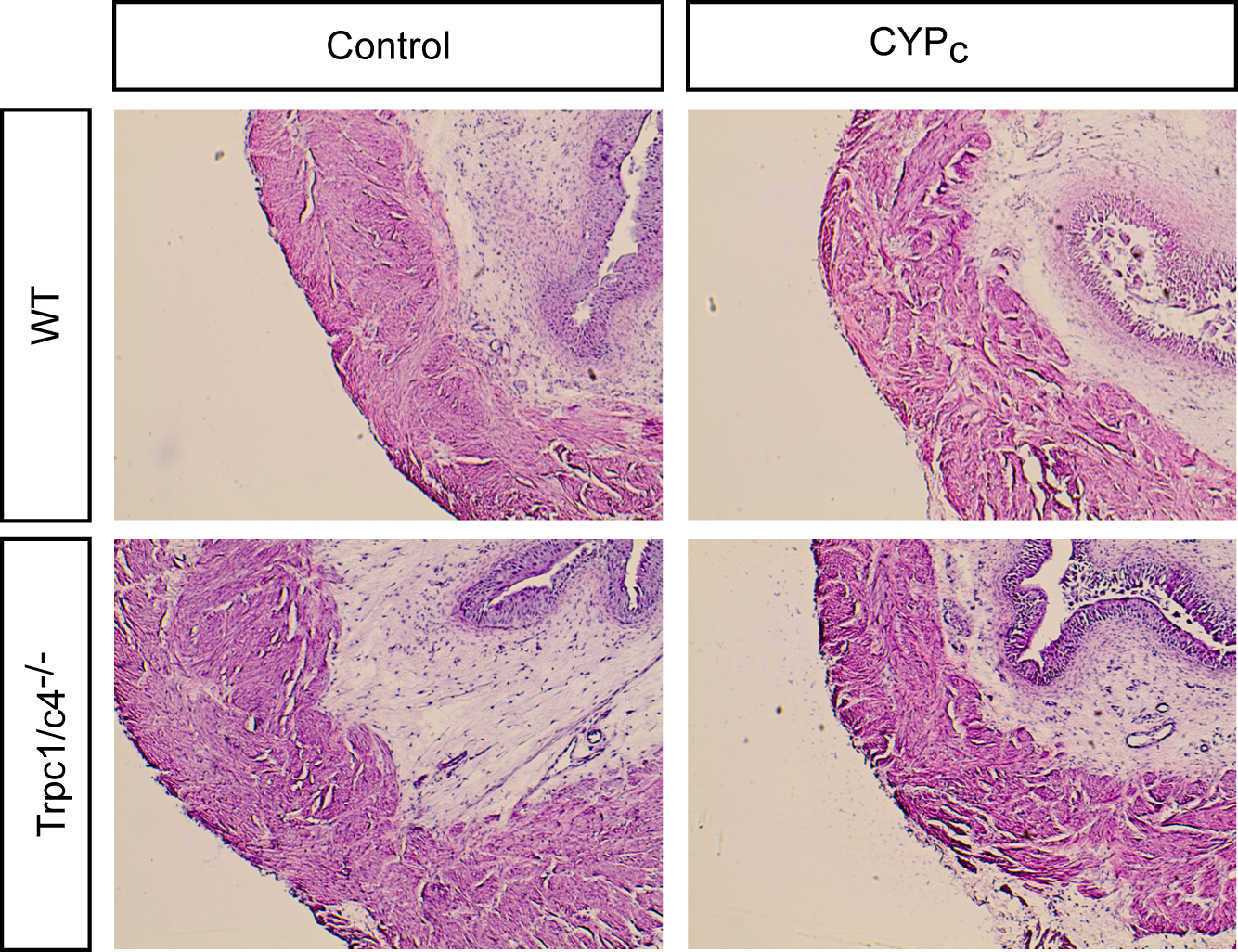

Supplement: Figure S1 — (TIF) [file pone.0069550.s001.tif]

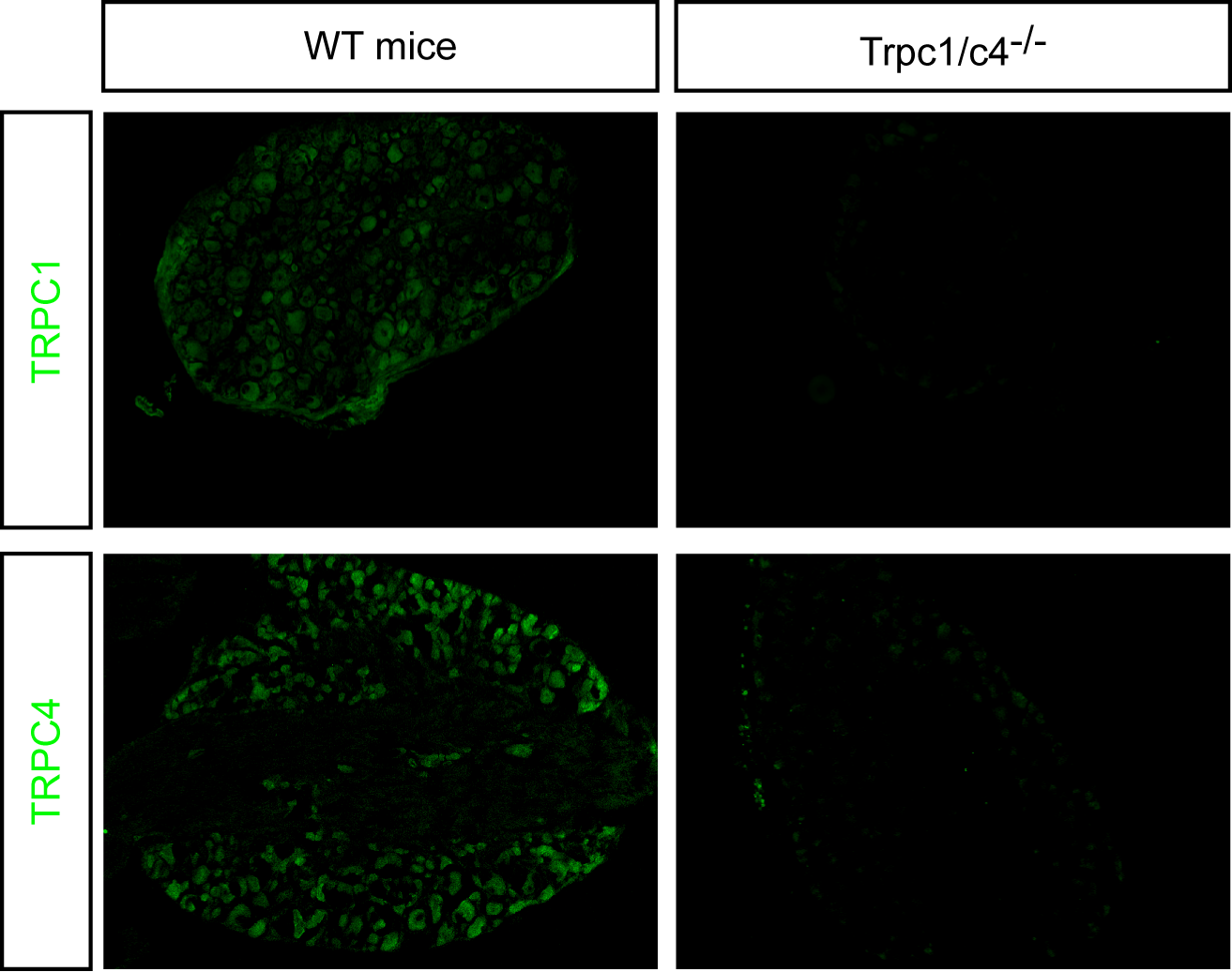

Supplement: Figure S2 — (TIF) [file pone.0069550.s002.tif]
